# Cesarean Section: A Potential and Forgotten Risk for Abdominal Wall Endometriosis

**DOI:** 10.7759/cureus.17410

**Published:** 2021-08-24

**Authors:** Patricia Ananias, Kanita Luenam, Joao Pedro Melo, Arunima Mariya Jose, Sayma Yaqub, Arifa Turkistani, Arpita Shah, Lubna Mohammed

**Affiliations:** 1 Family Medicine, California Institute of Behavioral Neurosciences & Psychology, Fairfield, USA; 2 Pathology, California Institute of Behavioral Neurosciences & Psychology, Fairfield, USA; 3 Internal Medicine, Sree Gokulam Medical College and Research Foundation, Trivandrum, IND; 4 Psychology, California Institute of Behavioral Neurosciences & Psychology, Fairfield, USA; 5 Public Health, University of Texas Health Science Center at Houston, Houston, USA; 6 Internal Medicine, California Institute of Behavioral Neurosciences & Psychology, Fairfield, USA; 7 Internal Medicine/Family Medicine, California Institute of Behavioral Neurosciences & Psychology, Fairfield, USA

**Keywords:** endometriosis and chronic pelvic pain, cutaneous endometriosis, endometriosis surgery, cesarean section (cs), caesarian scar, anterior abdominal wall lesion, clear cell cancer

## Abstract

Cesarean section endometriosis (CSE) can be caused by the iatrogenic deposition of endometrial cells, glands, and stroma during any time of the surgical procedure. It can be asymptomatic or, more frequently, resulting in chronic pain. Our article intends to provide more clinical information on CSE symptomatology, diagnosis, and preventive methods available in the literature, and discuss the malignancy transformation risk.

We performed a systematic review based on the Preferred Reporting Items for Systematic Review and Meta-Analysis guidelines. We included all types of study designs and selected only English articles from 2016 and forward. A total of 268 patients with abdominal wall endometriosis (AWE) were included in the final review; 260 women had CSE and eight women had endometriosis related to another gynecologic procedure.

Attention for suggestive symptoms during anamnesis and the presence of abdominal nodules close to the cesarean scar should raise suspicions of scar endometriosis. In addition, abdominal ultrasonography (USG), computed tomography (CT), magnetic resonance imaging (MRI), and fine-needle aspiration (FNA) biopsy can be helpful to differentiate from other conditions such as incisional hernias, suture granulomas, or malignant tumors. However, the final diagnosis and treatment is still the complete excision of the tumor. Therefore, additional studies on pathophysiology would help with new preventive methods and less invasive therapeutic options.

## Introduction and background

Endometriosis is characterized by the presence of functioning endometrial glands and stroma outside the uterine cavity. It is most commonly found in the pelvis, especially the ovary or pelvic peritoneum, but also can affect the fallopian tubes, bladder, sigmoid colon, and rectum [1]. Although rare, it can occur in extra-pelvic locations such as abdominal walls or viscera, skin, urinary system, and gastrointestinal or respiratory tracts [1,2]. Robert Meyer first described postoperative scar endometriosis in 1903, and it can be caused by the dissemination of endometrial tissue to the wound at any time of surgery [3]. The iatrogenic deposition of endometrial tissue can be found at the uterine scar, abdominal wall musculature, or most commonly in the subcutaneous tissue [4].

Abdominal wall endometriosis (AWE) can occur after procedures such as cesarean section (incidence of 0.03-0.4%) or even less common with hysterectomy, salpingostomy, episiotomy, amniocentesis, and laparoscopy surgeries [1,2]. Scar endometriosis usually manifests as a firm and palpable mass or lump associated with cyclic pain, which can cause chronic and cyclic lower abdominal discomfort in women [5,6]. However, it may not be easy to diagnose, especially when asymptomatic or localized in deeper sites [4,7]. The time between the first symptoms appearance and definitive diagnosis can be around 10 years [7], and differential diagnosis may include lipoma, hernia, suture granulomas, abscess, desmoid tumor, or malignancies [5,7]. The reason why some women develop scar endometriosis after cesarean while others do not is not fully understood. Although the seeding of endometriotic cells plays an important role, the whole process is complex and involves estrogen stimulation after the delivery, angiogenic growth factors, chronic inflammation, and altered immunity [2].

After the initial clinical assessment, diagnostic methods may be used in cases of uncertainty or to guide further management, such as abdominal ultrasonography (USG), computed tomography (CT), magnetic resonance imaging (MRI), and fine-needle aspiration (FNA) biopsy. Although, the definitive treatment and diagnosis is the surgical removal of the endometrial scar tissue with histopathological biopsy [5,8]. Malignant transformation of cesarean section (C-section) scar endometriosis is rare, presenting with high mortality and a survival rate of about 57% [7]. There are about 23 cases reported in the literature of transformation in clear cell carcinoma (CCC) of the abdominal wall [9]. However, the increase in C-sections worldwide may also account for an increase in the numbers of scar endometriosis and its incidence of clear cell carcinoma [10].

This systematic review aims to assess the main characteristics, diagnostic tools, and prevention methods for C-section scar endometriosis, decreasing its diagnostic delay and unnecessary diagnostics methods. In addition, this review aims to aware doctors and patients about its possible malignant transformation, raising suspicions for changes in scar appearance or presence of symptoms.

## Review

Methods

This systematic review followed the Preferred Reporting Items for Systematic Review and Meta-Analysis (PRISMA) 2009 guidelines. We did a thorough search on PubMed to identify relevant articles on April 14, 2021. The generic keywords used on the search were: “endometriosis” AND “c-section scar” OR “abdominal wall pain” AND “malignancy” (Table 1). Also were used on the search the relevant Medical Subject Headings (MeSH) terms with Boolean operators like “AND” and “OR”: “endometriosis/complications/diagnostic/diagnostic imaging/mortality/pathology/prevention and control/surgery,” “c-section/adverse effects,” and “Cicatrix/ etiology/pathology” (Table 2).

**Table 1:** Number of research articles for Medical Subject Headings (MeSH) terms

Keyword	Database	Total results	Last five years	Results after inclusion/exclusion applied	Full-text results
"Endometriosis/complications"[Majr] OR "Endometriosis/diagnosis"[Majr] OR "Endometriosis/diagnostic imaging"[Majr] OR "Endometriosis/mortality"[Majr] OR "Endometriosis/pathology"[Majr] OR "Endometriosis/prevention and control"[Majr] OR "Endometriosis/surgery"[Majr]	PubMed	9,967	1,685	1,533	1,499
"Cesarean section/adverse effects"[Majr]	PubMed	4,403	1,073	1,016	982
"Cicatrix"[Mesh] AND "Cicatrix/etiology"[Majr] OR "Cicatrix/pathology"[Majr]	PubMed	6,832	1,174	649	622

**Table 2:** Number of research articles for the researched keyword

Keyword	Database	Total results	Last five years	Results after inclusion/exclusion applied	Full-text results
Endometriosis	PubMed	29,464	6,571	3,781	3,724
Abdominal wall pain	PubMed	6,533	2,032	639	611
Cesarean-section scar	PubMed	2,739	786	564	537
Abdominal wall endometriosis	PubMed	708	214	103	93
Malignancy	PubMed	3,632,009	634,829	274,850	271,415

Inclusion/Exclusion Criteria

For this systematic review, we selected full-text studies published in 2016 and onwards. We included studies on humans, females, and English language articles.

Results

**Figure 1:**
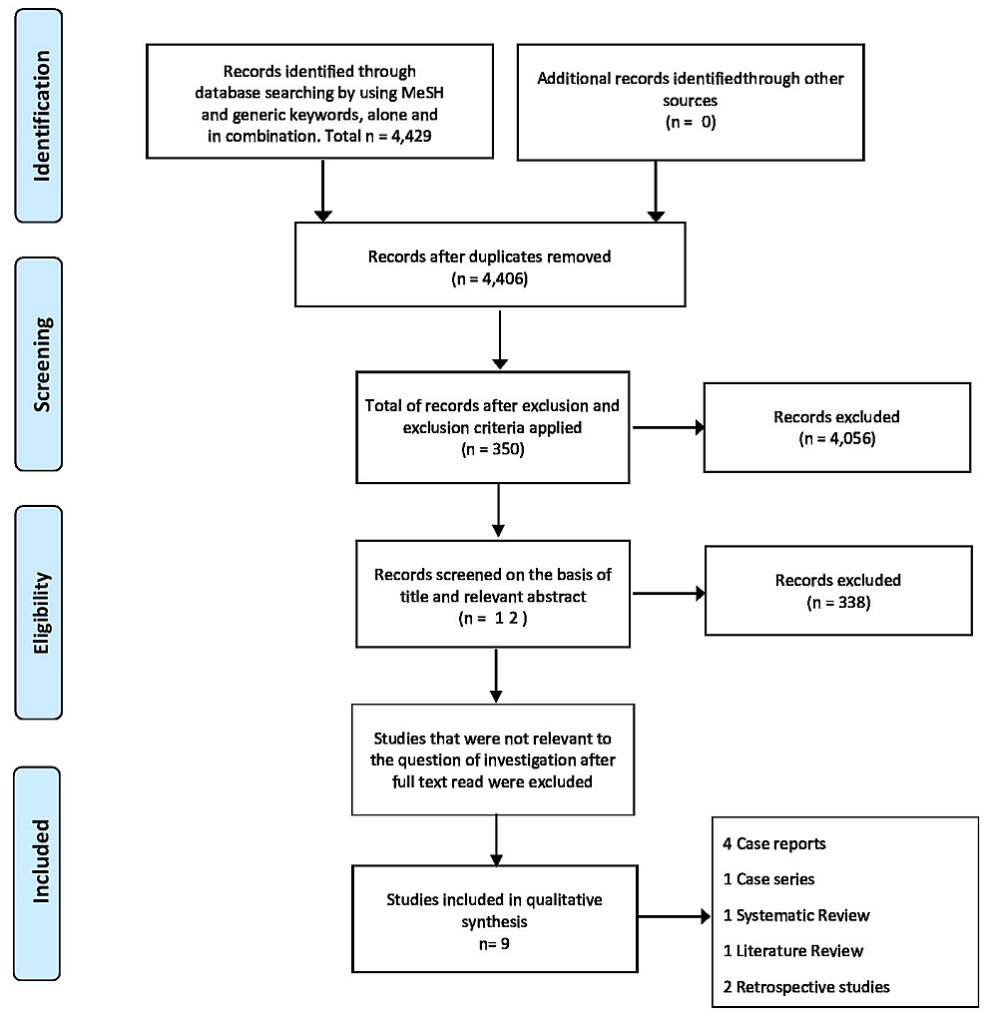
PRISMA flow chart showing the methodology PRISMA: Preferred Reporting Items for Systematic Reviews and Meta-Analysis.

A total of 4,429 studies were identified throughout database searching. We selected 350 papers after the removal of duplicates and applying the exclusion and inclusion criteria. Then, we screened each one of the articles based on the title and abstract. Next, we read the full text, and studies not relevant to the purpose of this study were removed; by the end, we were left with nine articles included in our review. Our review includes four case reports, one case series, two retrospective studies, one narrative review, and one traditional review (see Figure 1 with PRISMA 2009 flow diagram). A total of 268 patients with abdominal wall endometriosis were identified in those studies. A total of 260 patients had scar endometriosis related to previous cesarean section, and eight patients had scar endometriosis related to another gynecological procedure, such as hysterectomy. We included two case reports of malignant transformation of C-section scar endometriosis into clear cell carcinoma. Table 3 presents the studies selected for the review.

**Table 3:** Summary of reviewed articles AWE: abdominal wall endometriosis; CSE: cesarean scar endometriosis; C-section: cesarean section; CCC: clear cell carcinoma; USG: ultrasonography; MRI: magnetic resonance imaging; FNA: fine-needle aspiration.

Author	Year	Type of study	Patients	Purpose of the study	Conclusion
Carsote et al. [2]	2020	Literature review	_	Update on abdominal wall endometriosis (AWE) from a multinodal and multidisciplinary perspective.	There is an increasing incidence of AWE with the increase of gynecological/ obstetrical procedures, but the true prevalence in women is still not known. Clinical symptoms range from a lump to local pain. USG or MRI can be used for diagnosis, but surgical removal is curative and provides a definitive diagnosis.
D’Agostino et al. [1]	2019	Case report and literature review	8	Report of the 8^t^^h^subcutaneous case of pregnancy-related decidualization in post-cesarean endometriosis.	The hormonal changes in pregnancy can increase previous endometriotic nodules size and favor the development of other benign or malignant tumors.
Zhang et al. [6]	2019	Retrospective study	198	Identify the clinical features of cesarean scar endometriosis (CSE) and recommend precautionary measures.	Pfannenstiel incision carries a higher risk of CSE than the vertical midline incision. Preventive measures such as flushing and irrigating before closure could decrease the incidence of scar endometriosis.
Cocco et al. [7]	2019	Case report	2	Report two patients with AWE on subcutaneous and intramuscular localization. Their symptoms appeared a few years after C-section delivery.	USG can be used to confirm the diagnosis of abdominal wall endometriosis without the use of CT or MRI.
Mihailovici et al. [3]	2017	Systematic review	48	Clinical overview of endometriosis-associated malignant transformation in abdominal surgical scar.	Endometriosis-associated malignant transformation in the abdominal surgical scar is rare and aggressive, and clear cell histology has the worse prognosis. Clinicians should have a high level of suspicion for early diagnosis.
Fatima et al. [4]	2017	Case series	3	Report of two patients that developed AWE after cesarean and one patient after hysterectomy procedure.	Consider scar endometriosis as a differential diagnosis in women with a painful nodule or mass and a history of cesarean section or hysterectomy.
Song et al. [5]	2017	Retrospective review	7	Use of fine-needle aspiration (FNA) on the diagnosis of abdominal wall endometriosis.	The most common pelvic surgery associated with scar endometriosis was cesarean section (C-section), and FNA can be useful on diagnostic confirmation to guide further management.
Kostrzeba et al. [10]	2017	Case report	1	Report the development of clear cell carcinoma (CCC) of the abdominal wall in a patient 35 years after a C-section.	The increasing number of C-section deliveries may increase the number of patients with CCC of the abdominal wall.
Ferrandina et al. [9]	2016	Case report	1	Report a case of clear cell carcinoma arising from a CSE.	Attention should be given to CSE, especially when there are changes in the volume of nodules to rule out malignancy transformation.

Discussion

In 2015 about 21.1% of births, which correspond to 29.7 million, occurred through cesarean sections, almost twice the number performed in 2000 [11]. Based on this crescent rate, it is expected a subsequent increase in the diagnosis of endometriosis from C-section scar and a more significant occurrence of its transformation into malignancy [9]. The average time between the first complaints of symptoms and AWE's final diagnosis is about 10 years. Misdiagnosis or delay by clinicians can happen because symptoms can mimic other conditions [2,12]; patients may have their quality of life affected by being submitted to expensive and unnecessary diagnostic methods, leading to emotional and physical stress [7,13]. Thus, it is important to understand this condition by primary care physicians, surgeons, and dermatologists, as well as its typical and atypical presentations, pathophysiology, diagnosis, management, and malignancy risk [14,15].

Prevalence and Symptoms

Cesarean scar endometriosis has a very low incidence of 0.03-0.45% that can be explained due to its rarity or due to inconsistent epidemiological data reports [2]. Zhang et al.'s retrospective study described 198 pathologically confirmed cases of cesarean scar endometriosis (CSE), these patients had a mean age diagnosis of 32.0 ± 4 years, mean age at the time of C-section of 27.1 ± 3.5 years, a latency period from 1 to 120 months and in most of the cases they had only one CS (93.9%). The study also described Pfannenstiel incision as the most common incision found in CSE, and patients that had this type of incision presented a shorter latency period when compared with vertical midline incision. Although Pfannenstiel incisions have a better cosmetic appearance and decreased association of surgical hernias, they involve greater dissections of plans, more damage to the longitudinal abdominal capillaries, and consequently more blood loss, which can favor the implantation of endometrial cells at the edge of the operation cut that is difficult to be removed during the cesarean procedure [6].

Patients with CSE can be asymptomatic or present as a small, tender, and firm palpable nodule under or adjacent to a previous surgical incision [5,6,16]. A cyclic or non-cyclic pain can also be present and is usually the most frequent complaint [17]. The diagnosis should be suspected even when there is no evident history of endometriosis in all women of reproductive age, especially with a history of cesarean surgery [18]. Patients with the skin form of CSE can present with swelling or brownish to blood-like discharge from the lesion during their menses [17]. Pregnancy leads to hormonal and immunological changes, which can predispose to the development of tumors, either benign or malignant [1]. D’Agostino et al. describe the 8th case report of subcutaneous CSE that shows pregnancy-related decidualization, suggesting the inclusion of decidualization endometriosis among the differential diagnosis when changes in the size of a previous nodule or mass occur during pregnancy.

An atypical presentation of scar endometriosis that can mimic acute abdomen is the presence of premenstrual severe lower abdominal pain associated with intractable vomits, leading to dehydration and incapacity of taking anything orally. These cases can be managed conservatively during the acute phase with posterior excision of the endometrioma [19].

Diagnostic Methods

**Figure 2:**
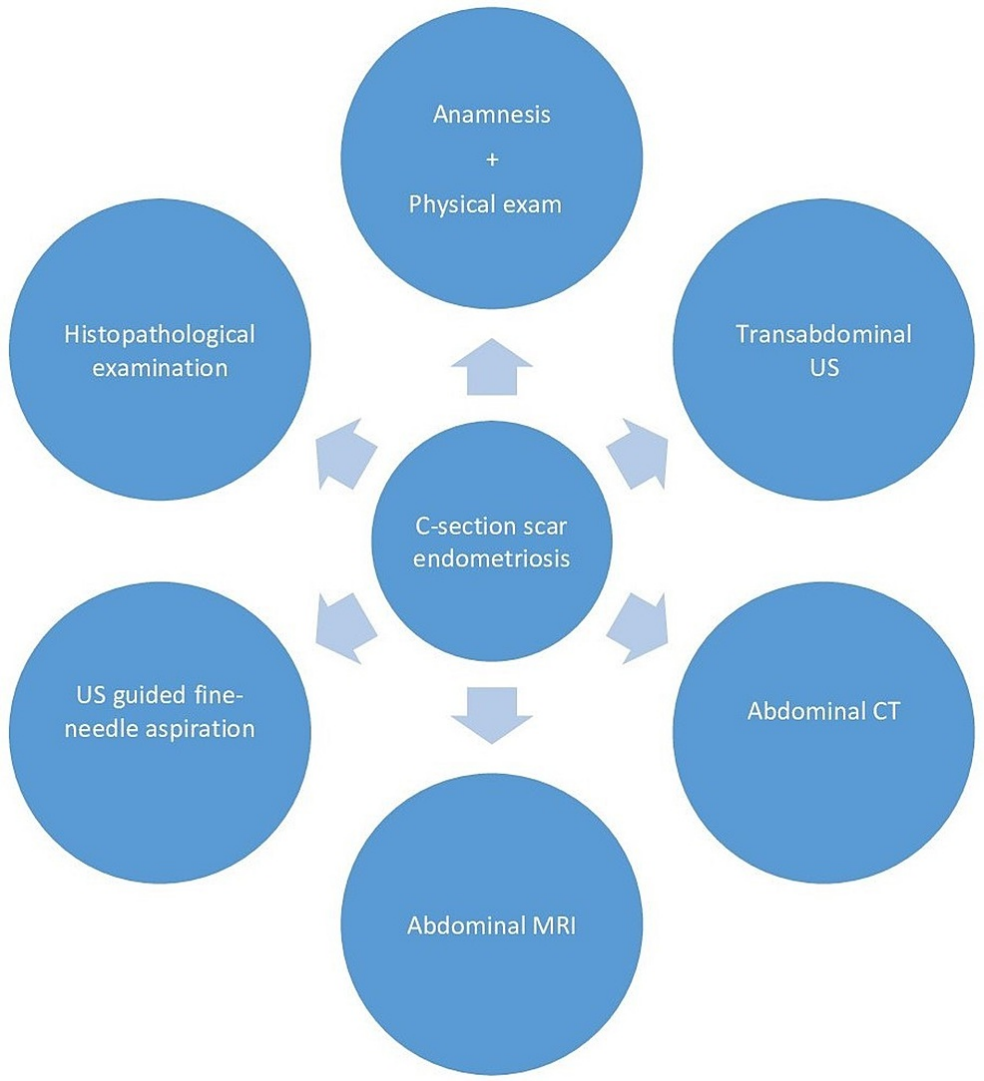
Diagram for cesarean section scar endometriosis diagnosis US: ultrasonography; CT: computed tomography; MRI: magnetic resonance imaging.

The clinical diagnosis can be made with the combination of suggestive symptoms and a physical exam associated with a history of surgical procedures [16]. However, when there are no classic symptoms of pain associated with menses, no discomfort or palpable nodules [4], assessment tools may be helpful to differentiate from other conditions such as lipoma, incisional hernia, suture granulomas, sebaceous cyst, neuroma, lymphadenopathy, and malignant tumors [5,13,16] (see Figure 2).

Transabdominal ultrasonography is the preferred method for initial investigation due to reduced cost, fewer side effects, and easy accessibility [7,8]. Ultrasonography (USG) helps differentiate solid, cystic, and heterogeneous masses and describes its relation to the skin and fascia [12,16]. On images, the endometriotic lesion appears as well defined, heterogeneous, hypoechoic mass with interior vascularization [4,7,20]. Cocco et al.’s study suggests that tenderness with USG probe pressure associated with a mass located close to the surgical scar increases the risk for diagnosis of AWE. Furthermore, Cocco et al. propose using a high-resolution linear probe with color-power Doppler (for the vascular pattern) and sonoelastography (to measure the stiffness of the mass) as essential tools for this diagnostic image method.

If the diagnostic is still unclear after assessment with ultrasound, CT or MRI may be used [2]. The abdominal CT shows a solid enhancing mass in a location related to a surgical site, and it is better to detect lesions on muscle or subcutaneous layer. While MRI shows a heterogeneous lesion with hyperintensity on T1 and T2-weighted images with or without sites of contrast enhancement, it is superior to detecting small lesions, detecting signs of hemorrhage, and presurgical evaluation [2,4].

Ultrasound-guided fine-needle aspiration (FNA) has also been used as an important diagnostic tool because it is not invasive, has a very low risk to disseminate cells, and can be performed without difficulties in palpable lesions [2]. The diagnostic criteria involve the presence of two of the following: endometrial glands, stromal cells, and hemosiderin-laden macrophages. When the glandular cells have atypical features (e.g., increased nuclear size, hyperchromasia, and irregular chromatin distribution), malignant transformation is possible and requires biopsy for further histologic evaluation [5,21]. Song et al. suggest that atypia is more commonly found in air-dried Diff-Quick stained smear than in Papanicolaou stained smear, and examiners should be aware of that to decrease the number of false positives on those preparations.

All these diagnostic tools help to suggest a diagnosis; however, only histopathological examination after removing the lesion can confirm the diagnosis showing the presence of ectopic endometriotic cells, glands, stroma, and hemosiderin-laden macrophages [8].

Pathogenesis and Prevention of AWE

The presence of pelvic endometriosis at the same time as abdominal wall endometriosis diagnosis has a variable rate; it can range from 0-25%, which is comparable to the presence of non-surgical endometriosis in the general population, therefore not relevant [22]. There is an experimental study conducted by Ridley and Edward, in which endometrial tissue is injected into the abdominal wall, and the cells proliferate into endometriotic foci [22]. This experiment supports the theory of iatrogenic seeding: during the cesarean section, when the uterus is opened, amniotic fluid can easily transport cells into the corners of the incisions at muscles, subcutaneous, and epidermis, and they can develop with the appropriate nutritional and hormonal environment [17]. The latency period is usually the time for the implanted cells to proliferate and organize, and when they reach a certain size, they cause localized pain during the menstrual cycle [8]. Zhang et al. showed that 64,6% (which corresponded to 135 patients) had endometriomas between the adipose and fascia layer, and 14.8% (31 patients) were localized between the adipose and muscular layer.

**Figure 3:**
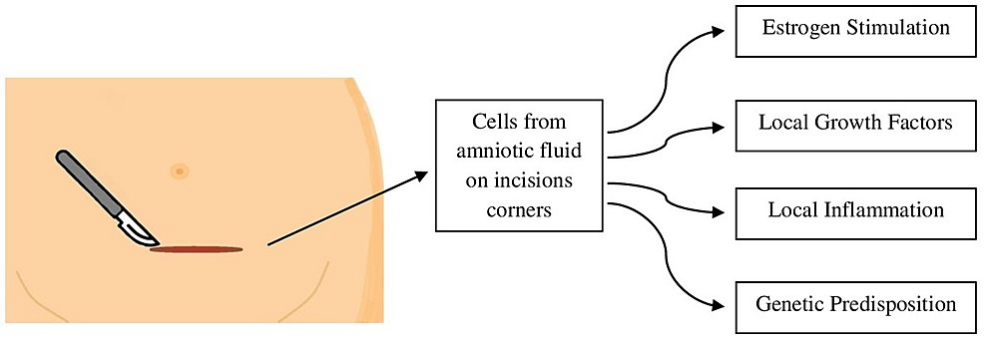
Pathogenesis for cesarean scar endometriosis

Carsote et al. describe a more complex explanation for AWE involving endocrine, immune, genetic, and inflammatory mechanisms. The local environment that predisposes CSE includes levels of estrogen (endometrial and stromal cells express high levels of estrogen receptors), growth factors able to activate inflammation, and metalloproteinases [2]. Moreover, non-surgical endometriosis is considered hereditary, and patients with first degree relatives have a risk of 6-9% higher of developing the disease [23], possibly due to genetic and epigenetic changes in endometrial cells, which may also be involved in AWE; however, more studies are necessary [2]. Figure 3 demonstrates the proposed mechanisms involved in the CSE pathogenesis.

Karapolat et al. discuss preventive surgery strategies for CSE. One of them is the use of abdominal compresses as physical barriers, placed on the skin and subcutaneous tissues before the opening of the uterus, thus protecting the incision margins. Another measure is to avoid the reuse of surgical tools (forceps, needle holders, suture materials, sponges) from the closure and cleaning of the uterus at the layers (skin, subcutaneous, fascia, and muscle) [8,16]. And lastly, the application of extensive irrigation with saline solution before the closure. These are speculative measures to prevent iatrogenic seeding and decrease scar endometriosis risk [8].

Malignancy Association/Management

Endometriosis-associated malignant transformation is rare; about 1 % of the endometriosis cases have developed into a malignancy, most of which happened at the ovary [9]. Clear cell carcinoma histology is the most commonly found, followed by endometrioid carcinoma [3]. There are 23 cases of CCC arising from endometriotic scars reported in the literature [9], and it tends to have poor outcomes compared to non-CCC tumors [3]. The tumor size is also important for prognosis; the outcome tends to be better when the mass is 4 to 9 cm in diameter. Furthermore, when the diagnosis is delayed, these tumors can reach very large proportions, up to 22 cm, adding another complication to the definitive treatment: the surgical excision may cause an important abdominal wall defect requiring a reconstruction surgery [9].

There are four criteria for the diagnosis of malignancy arising from endometriotic foci. The first three were proposed by Sampson in 1925, and the last one was proposed by Scott in 1953: (1) the tumor must have both malignant and benign endometrial tissues; (2) histology compatible with endometrial tissue; (3) exclusion of a second malignancy; and (4) presence of transitional morphology from benign to malignant endometriosis [3,24].

In Mihailovici et al.’s systematic review, CA-125 levels were measured before surgery in 21 cases; however, they were elevated in only nine cases and normal in the remaining 12 cases. Its analysis also did not found any specific marker for the endometriosis malignant transformation. There are no standardized guidelines for definitive treatment, and they are frequently based on each case presentation [25].

The potential benefit from using hormones as gonadotropin-releasing hormone (GnRH) or progestin before surgery is still unclear [3]. Patients with CCC scar endometriosis usually undergo radical resection of the tumor, and very often, they require partial or total removal of abdominal muscles [9,10], associated with hysterectomy, bilateral salpingo-oophorectomy, and inguinal/pelvic lymphadenectomy [9]. The most frequent adjuvant therapy found in the literature is platinum-based chemotherapy combined or not with radiation therapy [3]. Excision of large tumors may require the use of synthetic mesh to cover the abdominal wall defect. The material used must follow some criteria, including flexibility, strength, and not inducing allergic reactions, infections, or cancer. On the other hand, nets may also increase the risk of abdominal hernia, which can occur in 12-50% of the cases [10].

Limitations

We only analyzed articles published in the last five years in the English language. We can add as a weakness the small number of final studies after the screening process and the limited number of documented cases of CSE and CCC, which can result from the inconsistent epidemiological data report.

## Conclusions

Given the growing number of cesarean deliveries, the awareness of scar endometriosis among clinicians and patients is important as a possible late complication and cause of chronic pain in reproductive-age women submitted to cesarean section. Usually, patients present with a suggestive anamnesis of cyclic pain associated with a tender palpable mass close to a surgical scar, but atypical presentations and non-palpable masses may delay the final diagnosis. Diagnostic exams, when appropriate, are helpful to differentiate it from other conditions, especially when a malignant tumor is suspected. The use of abdominal compresses on the skin and subcutaneous tissue, the use of separate tools on the closure of uterus and abdominal layers, and irrigation with the saline solution could decrease the risk of seeding endometrial tissue on incisions. However, CSE parthenogenesis also involves endocrine, immune, inflammatory, and genetic factors that facilitate the growth of the ectopic implanted cells. Malignancy transformation is rare, but it poses a poor outcome, particularly when clear cell carcinoma and large tumors are found. Treatment of the malignant tumor is based on each case presentation, and large excisions may require a synthetic mesh to cover the abdominal wall defect. Additional studies on CSE pathophysiology would be needed to help with other preventive measures, evaluation of its efficacy, and a possible less invasive and expensive definitive treatment.

## References

[1] D'Agostino C, Surico D, Monga G, Palicelli A (2019). Pregnancy-related decidualization of subcutaneous endometriosis occurring in a post-caesarean section scar: case study and review of the literature. Pathol Res Pract.

[2] Carsote M, Terzea DC, Valea A, Gheorghisan-Galateanu AA (2020). Abdominal wall endometriosis (a narrative review). Int J Med Sci.

[3] Mihailovici A, Rottenstreich M, Kovel S, Wassermann I, Smorgick N, Vaknin Z (2017). Endometriosis-associated malignant transformation in abdominal surgical scar: a PRISMA-compliant systematic review. Medicine (Baltimore).

[4] Fatima K, Khanani S (2017). Scar endometriosis: an entity not to be forgotten. J Pak Med Assoc.

[5] Song SJ, McGrath CM, Yu GH (2017). Fine-needle aspiration cytology of endometriosis. Diagn Cytopathol.

[6] Zhang P, Sun Y, Zhang C, Yang Y, Zhang L, Wang N, Xu H (2019). Cesarean scar endometriosis: presentation of 198 cases and literature review. BMC Womens Health.

[7] Cocco G, Ricci V, Boccatonda A, Schiavone C (2020). Focused ultrasound for the diagnosis of non-palpable endometriotic lesions of the abdominal wall: a not-uncommon surgical complication. J Ultrasound.

[8] Karapolat B, Kucuk H (2019). A rare cause of abdominal pain: scar endometriosis. Emerg Med Int.

[9] Ferrandina G, Palluzzi E, Fanfani F (2016). Endometriosis-associated clear cell carcinoma arising in caesarean section scar: a case report and review of the literature. World J Surg Oncol.

[10] Kostrzeba E, Barczyk M, Wichtowski M, Garstecki R, Murawa D (2017). Clear cell carcinoma of the abdominal wall. Pol Przegl Chir.

[11] Boerma T, Ronsmans C, Melesse DY (2018). Global epidemiology of use of and disparities in caesarean sections. Lancet.

[12] Tatli F, Gozeneli O, Uyanikoglu H (2018). The clinical characteristics and surgical approach of scar endometriosis: a case series of 14 women. Bosn J Basic Med Sci.

[13] Yıldırım D, Tatar C, Doğan O (2018). Post-cesarean scar endometriosis. Turk J Obstet Gynecol.

[14] Burghaus S, Hildebrandt T, Fahlbusch C (2019). Standards used by a clinical and scientific endometriosis center for the diagnosis and therapy of patients with endometriosis. Geburtshilfe Frauenheilkd.

[15] Goel P, Devi L, Tandon R, Saha PK, Dalal A (2011). Scar endometriosis - a series of six patients. Int J Surg.

[16] Malutan AM, Simon I, Ciortea R, Mocan-Hognogi RF, Dudea M, Mihu D (2017). Surgical scar endometriosis: a series of 14 patients and brief review of literature. Clujul Med.

[17] Alnafisah F, Dawa SK, Alalfy S (2018). Skin endometriosis at the cesarean section scar: a case report and review of the literature. Cureus.

[18] Costa JE, Accetta I, Maia FJ, SÁ RAM (2020). Abdominal wall endometriosis: experience of the General Surgery Service of the Antônio Pedro University Hospital of the Universidade Federal Fluminense. Rev Col Bras Cir.

[19] Gaba N, Gaba S, Gupta M, Singla M, Dua A (2020). Endometriosis at cesarean scar presenting as an acute abdomen. Cureus.

[20] Draghi F, Cocco G, Richelmi FM, Schiavone C (2020). Abdominal wall sonography: a pictorial review. J Ultrasound.

[21] Ail DA, Joshi AR, Manzoor I, Patil S, Kulkarni M (2018). Fine-needle aspiration cytology of abdominal wall endometriosis: a meaningful adjunct to diagnosis. Oman Med J.

[22] Marras S, Pluchino N, Petignat P, Wenger JM, Ris F, Buchs NC, Dubuisson J (2019). Abdominal wall endometriosis: an 11-year retrospective observational cohort study. Eur J Obstet Gynecol Reprod Biol X.

[23] Koninckx PR, Ussia A, Adamyan L, Wattiez A, Gomel V, Martin DC (2019). Pathogenesis of endometriosis: the genetic/epigenetic theory. Fertil Steril.

[24] Grandi G, Toss A, Cortesi L, Botticelli L, Volpe A, Cagnacci A (2015). The association between edometriomas and ovarian cancer: preventive effect of inhibiting ovulation and menstruation during reproductive life. Biomed Res Int.

[25] Graur F, Mois E, Elisei R, Furcea L, Dragota M, Zaharie T, Al Hajjar N (2017). Malignant endometriosis of the abdominal wall. Ann Ital Chir.

